# Poly A- Transcripts Expressed in HeLa Cells

**DOI:** 10.1371/journal.pone.0002803

**Published:** 2008-07-30

**Authors:** Qingfa Wu, Yeong C. Kim, Jian Lu, Zhenyu Xuan, Jun Chen, Yonglan Zheng, Tom Zhou, Michael Q. Zhang, Chung-I Wu, San Ming Wang

**Affiliations:** 1 Center for Functional Genomics, Division of Medical Genetics, Department of Medicine, ENH Research Institute, Northwestern University Feinberg School of Medicine, Chicago, Illinois, United States of America; 2 Department of Ecology and Evolution, University of Chicago, Chicago, Illinois, United States of America; 3 Cold Spring Harbor Laboratory, Cold Spring Harbor, New York, United States of America; 4 Robert H. Lurie Comprehensive Cancer Center, Northwestern University Feinberg School of Medicine, Chicago, Illinois, United States of America; Wellcome Trust Sanger Institute, United Kingdom

## Abstract

**Background:**

Transcripts expressed in eukaryotes are classified as poly A+ transcripts or poly A- transcripts based on the presence or absence of the 3′ poly A tail. Most transcripts identified so far are poly A+ transcripts, whereas the poly A- transcripts remain largely unknown.

**Methodology/Principal Findings:**

We developed the TRD (Total RNA Detection) system for transcript identification. The system detects the transcripts through the following steps: 1) depleting the abundant ribosomal and small-size transcripts; 2) synthesizing cDNA without regard to the status of the 3′ poly A tail; 3) applying the 454 sequencing technology for massive 3′ EST collection from the cDNA; and 4) determining the genome origins of the detected transcripts by mapping the sequences to the human genome reference sequences. Using this system, we characterized the cytoplasmic transcripts from HeLa cells. Of the 13,467 distinct 3′ ESTs analyzed, 24% are poly A-, 36% are poly A+, and 40% are bimorphic with poly A+ features but without the 3′ poly A tail. Most of the poly A- 3′ ESTs do not match known transcript sequences; they have a similar distribution pattern in the genome as the poly A+ and bimorphic 3′ ESTs, and their mapped intergenic regions are evolutionarily conserved. Experiments confirmed the authenticity of the detected poly A- transcripts.

**Conclusion/Significance:**

Our study provides the first large-scale sequence evidence for the presence of poly A- transcripts in eukaryotes. The abundance of the poly A- transcripts highlights the need for comprehensive identification of these transcripts for decoding the transcriptome, annotating the genome and studying biological relevance of the poly A- transcripts.

## Introduction

The genome is expressed through transcription that generates different classes of RNA molecules, specifically, ribosomal RNAs, messenger RNAs, and small RNAs, which constitute the transcriptome content. The transcriptional process is regulated at multiple levels with differential promoter usage, alternative splicing, intron retention, and alternative polyadenylation etc. Furthermore, the abundance of individual transcripts can vary up to million-fold levels. Thus, the transcriptome is far more complicated than the original coding sequences in the genome, and decoding the transcriptome will be more challenging than decoding the genome.

Early studies identified the presence or absence of the 3′ poly A tail on transcripts, resulting in their classification as either poly A+ transcripts or poly A- transcripts [1]–[11]. This classification has been firmly confirmed by a recent genome tiling array study [12]. The poly A+ transcripts include mRNA, microRNA and snoRNA generated by RNA polymerase II [13]; the poly A- transcripts currently known include ribosomal RNAs generated by RNA polymerase I [14], histone RNAs generated by RNA polymerase II [15], and tRNAs and other small RNAs generated by RNA polymerase III [16].

An ultimate goal of transcriptome study is to identify all transcripts at the sequence level. This has been very successful for the poly A+ transcripts, largely attributed to the presence of 3′ poly A tail that facilitates their isolation and cDNA synthesis by using oligo dT. Up to now, millions of poly A+ transcripts have been sequenced from various species. Regardless of the evidence indicating the wide prevalence of poly A- transcripts, however, only a few poly A- transcripts have been identified so far at the sequencing level. Without the poly A- transcript information, the transcriptome complexity and genome organization cannot be fully understood.

The lack of poly A- transcript information is largely associated with the technical factors. Unlike the poly A+ transcripts that have the universal 3′ poly A tail, there is no known consensus sequence in poly A- transcripts for isolation and cDNA synthesis. To overcome this obstacle, we developed a technical system termed Total RNA Detection (TRD). The system consists of three key elements: 1) enriching the poly A- transcripts by depleting the abundant ribosomal and tRNA transcripts; 2) synthesizing cDNA without regard to the status of the 3′ poly A tail; and 3) using the 454 sequencer for massive 3′ EST collection [17]. The 3′ EST provides poly A signal and poly A tail information to distinguish between poly A+ transcripts and poly A- transcripts, can be compared directly with known transcripts, and can map to the genome with 3′ boundary location for the mapped locus. Using the TRD system, we analyzed the cytoplasmic transcripts of HeLa cells, a model cell line widely used for transcriptome study. Our sequence data indicate that the poly A- transcripts indeed exist.

## Results and Discussion

### Transcript enrichment and sequence collection

**Figure 1:**
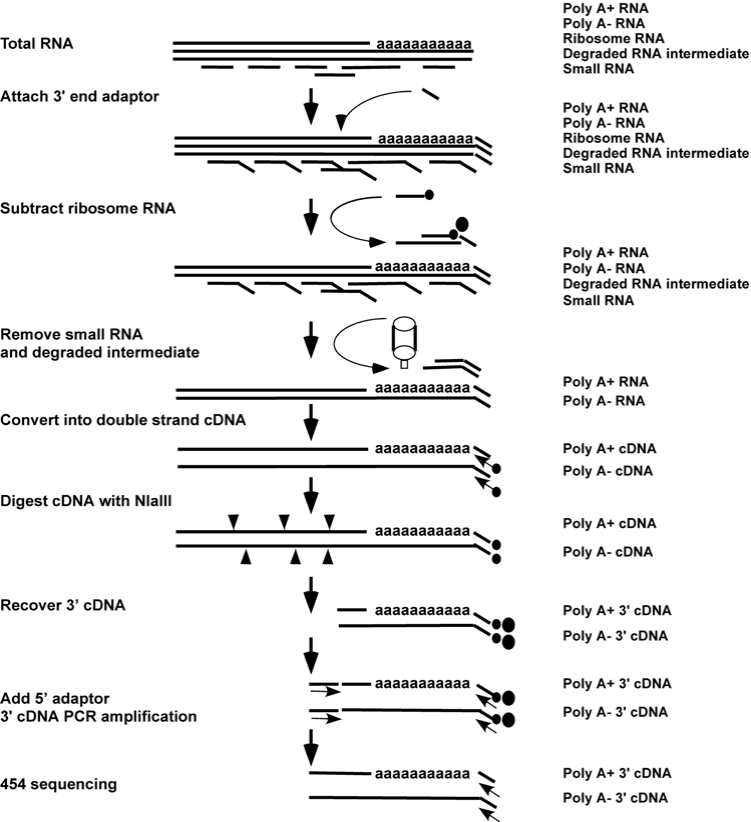
The Total RNA Detection system. A universal RNA adaptor was firstly added to the 3′ ends of all RNA templates. The abundant 18S and 28S ribosome RNAs were then subtracted by using biotinylated ribosomal-specific probes. Small-size RNAs containing the degraded RNA intermediates were removed by size-filtration. The enriched transcripts were converted into double-strand cDNA by using the 3′ end RNA adaptor-based primer. The cDNAs were further digested by NlaIII. The 3′ cDNAs were isolated by using the streptoavidin beads. An adaptor was added to the 5′ ends of the 3′ cDNAs. The 3′ cDNAs were then amplified by PCR using the 5′ adaptor-based sense primer and the 3′ end RNA adaptor-based antisense primer. The amplified 3′ cDNAs were sequenced from the 3′ end by the 454 system. See further details in Materials and Methods.

A challenge for studying the poly A- transcripts is to distinguish the true poly A- transcripts from the degraded transcripts originated from *in vivo* physiological RNA metabolism or *in vitro* RNase activities (which also lack the 3′ poly A tail). Another challenge is the scarcity of the poly A- transcripts in the total transcriptome content. Three key steps in the Total RNA Detection system were used to address these issues (Figure 1).

1. Adding a universal adapter to the 3′ end of all transcripts before processing the RNA sample. This adaptor protects the 3′ end of transcripts at the very beginning, provides a universal priming site for later cDNA synthesis, and serves as an identifier to distinguish between the sequences from the 3′ end of true transcripts and the sequences from experimental artifacts.
2. Removing ribosomal RNAs. Subtraction was applied to remove the abundant 28S and 18S ribosomal RNAs by using two specific probes for both 18S ribosomal RNA and 28S ribosomal RNA.
3. Removing small-size RNAs. Sizing exclusion was used to remove the small-size RNAs that include the abundant tRNAs, snoRNAs and the degraded transcript intermediates. Although this process will remove certain authentic small RNAs such as miRNA, these small RNAs have been extensively characterized by many other studies and they are not the focus of the study. Removal of small-size RNAs will provide a better background to identify the true poly A- transcripts.

The enriched transcripts were converted into cDNA by using the biotinylated primer based on the universal adaptor sequence at the 3′ ends of transcripts. The cDNA was then digested by a 4-base cutting restriction enzyme NlaIII to generate the 3′ cDNAs at about 140 bps [18] that fit with the 454 sequencing range. The 3′ cDNA was recovered by using streptoavidin beads. An adaptor was ligated to the 5′ end of the recovered 3′ cDNA. To generate 3′ cDNA templates for 454 sequencing, the 3′ cDNA was amplified by PCR using the sense primer based on the 5′ adaptor sequences and the antisense primer based on the 3′ end university adaptor seqeunces. 454 sequencing was performed from the 3′ ends of the cDNA. A total of 273,949 raw sequences was collected by a single 454 sequencing run. After removing sequences from remaining ribosomal RNAs, tRNAs, mitochondrial RNAs, sequences without a 3′ end adaptor, and sequences shorter than 11 bp, 148,520 sequences were qualified, representing 52,571 distinct 3′ ESTs. Of the 52,571 3′ ESTs, 13,782 were mapped to the human genome reference sequences (HG18) and were used for the following analyses (Table 1, Table S1).

**Table 1:** Summary of the sequence information.

Items	Number (%)
Total sequences	273,949
With 3′ end tag	241,864 (100)
28S ribosome RNA	29,049 (12)
18S ribosome RNA	43,831 (18)
5.8S RNA	346 (0.1)
5S RNA	17 (0)
tRNA	1,228 (0.5)
Mitochondrion RNA, <11 bps, low quality	18,783 (8)
Final qualified sequences*	148,520 (61)
Final distinct sequences	52,571
Genome-mapped sequences	13,782

* After removing sequences of 28S, 18S, 5.8S, 5S, tRNAs, mitochondrial RNA, <11 bps, and low quality.

### Classification, novelty, and abundance of 3′ ESTs

Based on the factors of poly A signal, poly A tail and matching to known transcript sequences, standards were set to classify the 3′ ESTs into three subgroups:

#### Poly A- 3′ EST

A sequence with no or only one A at the 3′ end (A accounts for 1/4 probability at this position out of A, G, C, and T) AND without a poly A signal AND matched to none of the known RefSeq/mRNA/EST/SAGE tags (nearly all known sequences are from poly A+ transcripts). The only known poly A- transcripts, which are histone transcripts, were determined by direct matching to known histone mRNA sequences.

#### Poly A+ 3′ EST

A sequence matched to either the RefSeq/mRNA/EST/SAGE tags and with one or more A at the 3′ end with/without a poly A signal, OR a sequence matched to none of the known RefSeq/mRNA/EST/SAGE tags (novel) but with one A and a poly A signal or more As with/without a poly A signal.

#### Bimorphic 3′ EST

A sequence matched to either the RefSeq/mRNA/EST/SAGE tags and with no A at the 3′ end with/without a poly A signal, OR a sequence matched to none of the known RefSeq/mRNA/EST/SAGE tags (novel) and with no A at the 3′ end but with a poly A signal. The bimorphic 3′ EST represents the isoform of the poly A+ transcript. Its lack of the 3′ poly A tail reflects the dynamics of poly A tail metabolism in the poly A+ transcript.

Poly A signals were defined in the order of prevalence: AATAAA, ATTAAA, TATAAA, AGTAAA, AAGAAA, AATATA, AATACA, CATAAA, GATAAA, AATGAA, TTTAAA, ACTAAA, AATAGA [19].

Based on the standards, 24% of 3′ ESTs (2% are histone 3′ ESTs) were classified as poly A- 3′ EST, 36% as poly A+ 3′ EST, and 40% as bimorphic 3′ EST (Table 2, Table S1A, S1B, S1C). Most of the poly A+ and bimorphic 3′ ESTs match to known human transcript sequences that are basically from poly A+ transcripts. The unmatched novel poly A+ 3′ ESTs tend to have short poly A tails (Table S2A, S2B). Although the poly A- 3′ EST accounts for 24% of total sequences, they only contribute 12% of the total sequence copies (Table 2), confirming that the poly A- transcript is highly heterogeneous but expressed at lower levels [1], [12]. Similarly, the bimorphic 3′ EST accounts for 41% of the total sequences but only contributes 15% of the total sequence copies. The lack of high-copy bimorphic 3′ EST implies that the highly expressed poly A+ transcripts may not use the change of poly A length as a regulatory mechanism. The poly A+ 3′ EST accounts for 36% of total sequences but contributes 73% of the total copies, indicating that the poly A+ transcript contributes far more abundance to the transcriptome than the poly A- and bimorphic transcripts.

**Table 2:** Novelty, classification and abundance distribution of 3′ EST.

	Total (%)	Classification		
		Poly A-	Poly A+	Bimorphic
Total	13,782 (100)	3,299 (24)	4,898 (36)	5,585 (40)
Compare to known mRNA				
Non match	4,086 (30)	2,984 (22)	924 (7)	178 (1)
Match	9,696 (70)	315 (2)*	3,974 (29)	5,407 (39)
RefSeq	1,629	0	647	982
mRNA	3,149	0	1,272	1,877
EST	6,350	0	2,732	3,618
SAGE tag	4,727	0	1,951	2,776
Abundance distribution				
Total copies	55,172 (100)	6,369 (12)	40,317 (73)	8,486 (15)
>1000	5	0	5	0
501 to 1000	6	1	5	0
101 to 500	22	6	15	1
51 to 100	26	6	18	2
11 to 50	153	24	110	19
6 to 10	235	28	133	74
2 to 5	3,084	596	1,241	1,247
1	9,936	2,323	3,371	4,242

* The 315 3′ ESTs matched to histone sequences.

**Figure 2:**
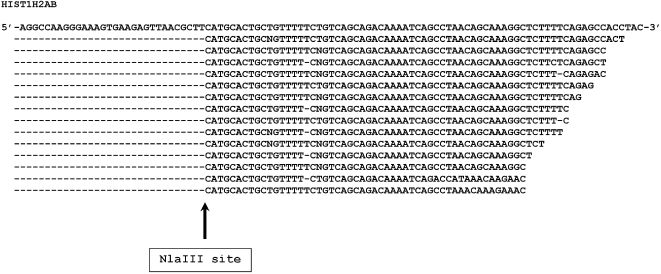
Example of histone 3′ EST distribution. Fifteen 3′ ESTs that map to the full-length histone 1H2AB cDNA sequences (NM_003513) are clustered proximal to the 3′ end of the full-length sequence. See Table 3, Table S3 and Figure S1 for the distribution of other histone 3′ ESTs.

Histone transcripts are the only known polymerase II-generated poly A- transcripts with sequence information [12], [15], [20]. A total of 315 distinct 3′ ESTs from 46 histone genes was detected in this study. Comparison of the 3′ ends of the detected histone 3′ ESTs to the full-length histone transcript sequences shows that 80% of the 3′ ESTs matched proximal to the 3′ end of their corresponding full-length sequences (Table 3, Figure 2, Figure S1, Table S3). The pattern of the 3′ end distribution of these histone sequences closely resembles that observed in a recent histone study [21]. The high-degree of intact 3′ ends of the detected histone transcripts provides an internal control for the authenticity of the poly A- transcripts detected in this study.

**Table 3:** The 3′ end distribution of histone 3′ ESTs.

Histone mRNA	Number of matched 3′ ESTs	Match to 3′ end of histone mRNA	
		Distal to 3′ end	Proximal to 3′ end
HIST1H1B	4	2	2
HIST1H1C	3	3	
HIST1H1E	1		1
HIST1H1T	2		2
HIST1H2AB	15	1	14
HIST1H2AC	1		1
HIST1H2AE	1		1
HIST1H2AG	3	3	
HIST1H2AI	2		2
HIST1H2AJ	1		1
HIST1H2AK	3	2	1
HIST1H2AM	1		1
HIST1H2BB	2		2
HIST1H2BC	1		1
HIST1H2BD	8	1	7
HIST1H2BE	1		1
HIST1H2BF	2		2
HIST1H2BG	10	3	7
HIST1H2BH	4	2	2
HIST1H2BJ	1		1
HIST1H2BN	2		2
HIST1H3A	7	2	5
HIST1H3B	20	2	18
HIST1H3C	5		5
HIST1H3D	16	1	15
HIST1H3E	1		1
HIST1H3F	29		29
HIST1H3G	23		23
HIST1H3H	18	1	17
HIST1H4A	8		8
HIST1H4B	8	1	7
HIST1H4C	26		26
HIST1H4E	8	5	3
HIST1H4H	2		2
HIST1H4I	9		9
HIST1H4J	11	4	7
HIST1H4K	4		4
HIST2H2AA	9	4	5
HIST2H2AC	5		5
HIST4H4	9	8	1
H1FX	1		1
H2AFX	7	7	
H2AFY	1	1	
H3F3A*	8	6	2
H3F3B	1	1	
H4/o	11	3	8
Total (%)	315 (100)	63 (20)	252 (80)

* H3F3A mRNA is polyadenylated (Wells D, Kedes L. PNAS 82, 2834, 1985)

### Map 3′ ESTs to the human genome reference sequences

We analyzed the genome origins for the 8,178 3′ ESTs that mapped to a single location in the genome. Nearly two-thirds of the 3′ ESTs mapped to the intragenic region and a third to the intergenic region (Table 4, Table S1A, S1B, S1C). The poly A- 3′ EST maps more to the intergenic regions than poly A+ and bimorphic 3′ ESTs. Of the 3′ ESTs mapped to the intragenic region, most mapped to the introns, and the poly A- 3′ EST orients more in the antisense direction. Those mapped to the intron regions might represent the transcripts that are the intron-retained isoforms of the annotated genes, or the transcripts that are from the genes overlapping with the annotated genes, or the alternatively spliced transcripts in which their last exon sequences differ due to alternative splicing, or the transcripts that are largely originated from the intron regions with regulatory function such as the microRNA [22]. Indeed, 491 intron-mapped 3′ ESTs mapped to 52 of the 219 known intron-originated microRNA precursors, 38 of which were matched by a single type of 3′ EST and 14 of which were matched by more than one type of 3′ EST (Table S4A, S4B). We compared the mapped loci by the poly A-, poly A+, and bimorphic 3′ ESTs. Using the average gene density of 75 kb in the human genome as a cut-off (40,007 “genes” in the human genome/3 Gb human genome size = 75 kb. http://www.ncbi.nlm.nih.gov/projects/Gene/gentrez_stats.cgiSNGLTAX9606), the results show that half of the mapped loci between two or three subtypes of 3′ ESTs do not overlap each other (Table 5, Table S5A, S5B1-3, S5C1-3), suggesting that many different subtypes of transcripts are transcribed from different genomic loci.

**Table 4:** Mapping 3′ ESTs to the human genome reference sequences (HG18).

	Total (%)	Poly A-	Poly A+	Bimorphic
Total mapped 3′ EST	13,467	2,984	4,898	5,585
Single mapped 3′ EST	8,178 (100)	2,113 (100)	2,760 (100)	3,305 (100)
Intergenic mapping*	2,310 (28)	808 (38)	751 (27)	751 (23)
Intragenic mapping	5,868 (72)	1,305 (62)	2,009 (73)	2,554 (77)
Distribution of Intragenic mapping				
Total	5,868 (100)	1,305 (100)	2,009 (100)	2,554 (100)
Sense	5,426 (93)	1,123 (86)	1,871 (93)	2,432 (95)
*intron*	4,182	976	1,385	1,816
*exon*	1,125	125	441	558
*exon/intron*	127	22	45	58
Antisense	442 (8)	182 (14)	138 (7)	122 (5)
*intron*	385	175	113	95
*exon*	24	5	5	13
*exon/intron*	37	2	20	14

* The mapping difference is at statistically significant level (p = 3.67×10 ^−16^, X^2^ test).

**Table 5:** Overlapping genome position of 3′ ESTs.

Type of overlapping	Type of sequences (%)		
	Poly A+	Poly A-	Bimorphic
Common in 3	633 (23)	474 (22)	749 (23)
Common in 2*	832 (30)	468 (22)	898 (27)
Poly A+/Poly A-	481	471	
Poly A-/bimorphic		541	576
Poly A+/bimorphic	1,028		1,059
Only in 1	1,295 (47)	1,171 (55)	1,658 (50)
Total	2,760 (100)	2,113 (100)	3,305 (100)

* Multiple overlapping exists for the sequences of common in 2.

The intergenic loci mapped by the 3′ ESTs represent novel transcribed regions in the genome. To investigate the potential functional relevance, those sequences were compared with the genome sequences of 16 species to define their evolutionary conservation. The results show that of the 2,310 mapped intergenic sequences, 1,344 (59%) are conserved across different species, and the conservation rate for poly A- 3′ EST-mapped sequences is similar to the rates of the poly A+ and bimorphic 3′ EST-mapped sequences (Table 6, Table S6A, 6B, 6C).

**Table 6:** Evolutionary conservation of the 3′ EST-mapped intergenic regions*

Species	Divergence		Total sequences**	Type of sequences		
	Mean	Median	(*p*<0.05)	Poly A-	Poly A+	Bimorphic
Chimp	0.013	0.000	1,311	451	423	426
Macaque	0.056	0.042	1,263	436	399	418
Mouse	0.359	0.339	441	252	303	336
Rat	0.367	0.342	425	240	297	321
Rabbit	0.298	0.274	532	236	284	304
Dog	0.267	0.245	759	338	355	375
Cow	0.269	0.240	733	317	334	371
Armadillo	0.255	0.234	568	210	263	295
Elephant	0.254	0.227	600	222	276	301
Tenrec	0.328	0.304	404	192	242	266
Opossum	0.431	0.412	250	139	197	233
Chicken	0.388	0.342	130	52	105	104
Frog	0.364	0.297	77	31	49	58
Zebrafish	0.454	0.368	49	28	36	45
Tetraodon	0.455	0.360	43	34	31	38
Fugu	0.489	0.422	34	28	26	44
Total non-redundant 3′ EST (%)			1,322 (100)	467 (32)	428 (32)	437 (33)

* 2,310 3′ ESTs mapping to single intergenic region were used for the study

** p value was not used for chimp and macaque as they are too close to the humans.

### Confirmation of the 3′ ESTs

Four approaches were used to confirm the detected 3′ ESTs.

**Figure 3:**
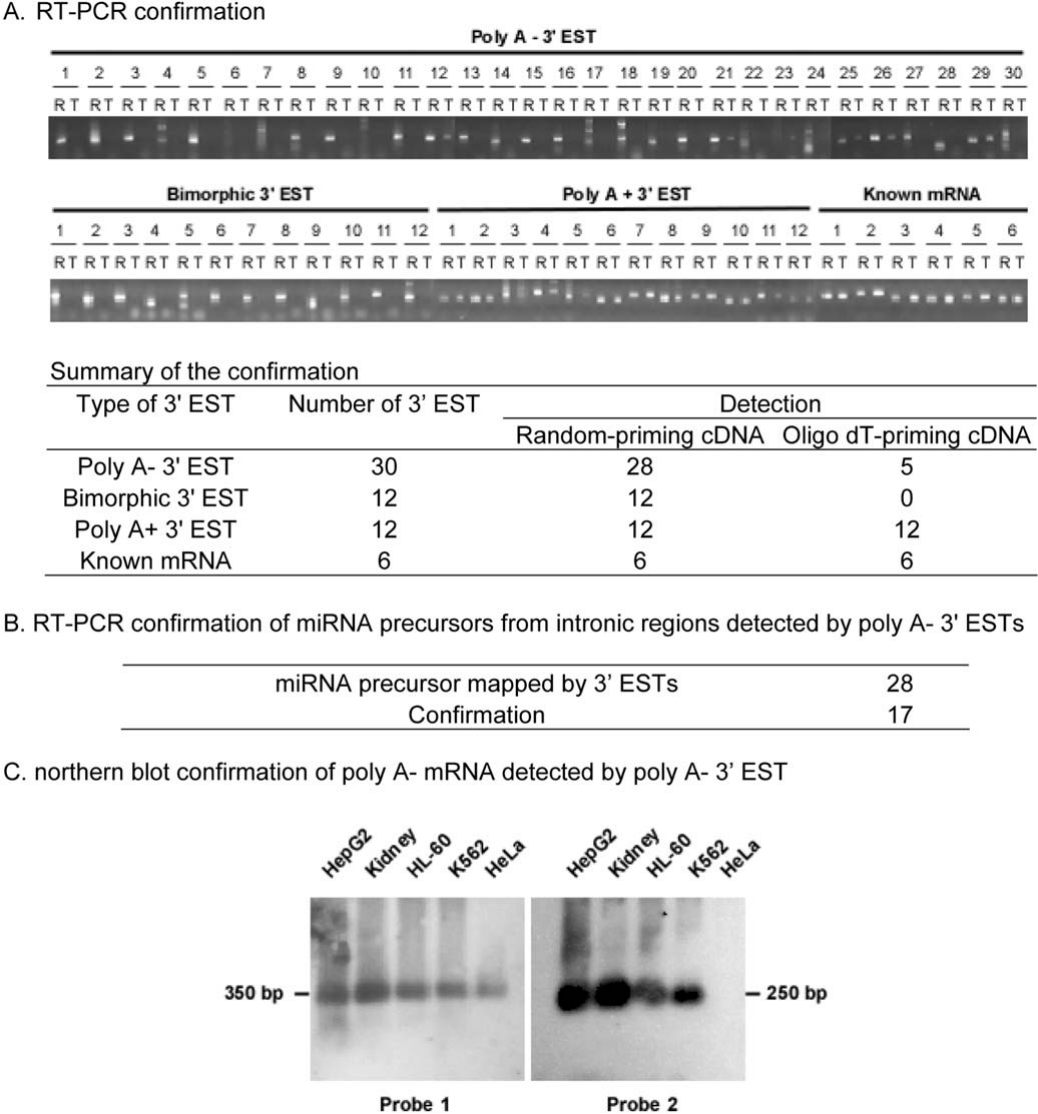
Experimental verification of novel 3′ ESTs. (A). 3′ end verification for each subtype of 3′ EST. 3′ ESTs from poly A-, poly A+, and bimorphic subtypes were selected for the confirmation. Known poly A+ transcripts were used as positive control. Random priming- generated cDNA and oligo dT-generated cDNA were used as the templates. R: cDNA generated by random priming; T: cDNA generated by oligo dT priming. See Table S7 for primer information. (B). Verification of 3′ ESTs mapped to intronic microRNA precursors. RT-PCR was used to verify the 3′ ESTs that map to intronic microRNA precursors. Amplified products were cloned and sequenced. See Table S4C for primer information. (C). northern blot verification of poly A- 3′ EST. Two poly A- 3′ ESTs were used as the probes (Table S7) and RNAs from five human cell lines were used for the detection.

1. RT-PCR was used to verify each subtype of novel 3′ ESTs. Two types of cDNAs were used as the templates. One was generated by random priming that does not rely on the poly A tail for cDNA synthesis, and the other was generated by oligo dT priming that relies on the poly A tail for cDNA synthesis. For poly A- transcripts, only the random-priming cDNA but not the oligo dT priming-cDNA should generate positive amplification; for bimorphic transcripts, the random-priming cDNA should and the oligo dT priming-cDNA could generate positive amplification; for poly A+ transcripts, both random-priming cDNA and oligo dT-priming cDNA should generate positive amplification. The results show that for the 28 positively detected poly A- 3′ ESTs, 23 were only detected in random-priming cDNA, confirming that their original transcripts do not have poly A tails; for the 12 bimorphic 3′ ESTs, 12 were detected only in random-priming cDNA, confirming that their original transcripts lack poly A tails; for the 12 poly A+ 3′ ESTs, all were detected in both random-priming cDNA and oligo dT-priming cDNA, confirming that their original transcripts have poly A tails (Figure 3A, Table S7).
2. RT-PCR was used to detect the 3′ EST-matched microRNA precursors that are transcribed from the intronic regions. For the 28 reactions, 17 were confirmed by sequencing the amplified products (Figure 3B, Table S4C).
3. northern blot was performed to verify the poly A- transcripts using poly A+ transcripts-depleted RNA samples from five human cell lines. Two poly A- 3′ ESTs that were verified to be originated from poly A- transcripts were used as the probes (Table S7). One probe detected signals in all five cell lines, and the other probe detected signals in four but not in HeLa cells, likely due to its low abundance in HeLa cells that was under the threshold of northern blot detection (Figure 3C).
4. The poly A- 3′ ESTs were compared with the poly A- “transfrag” detected in HepG2 cells by the genome-tiling array study [12]. For the 579 poly A- 3′ ESTs mapped to the 10 chromosomes covered by the array study, 210 overlapped with the poly A- “transfrags”, of which 37 (17%) to cytosolic, 53 (25%) to nuclear, and 120 (57%) map to both cytosolic and nuclear “transfrags” (Table 7, Table S8A, 8B, 8C). The high rate of overlapping in “both cytosolic and nuclear” part indicates that the poly A- transcripts are prevalent in both cytosolic and nuclear compartments.

**Table 7:** Poly A- 3′ EST overlapped with poly A- “transfrag”

Compartment	Type of sequences (%)	
	“Transfrag”	Poly A- 3′ EST
Cytosolic	6,185 (14)	37 (18)
Nuclear	17,995 (41)	53 (25)
Both cytosolic and nuclear	20,200 (46)	120 (57)
Total	44,380 (100)	210 (100)

A total of 52,571 distinct sequences were identified from the raw sequences, of which only 13,782 mapped to the human genome reference sequences. For these unmapped sequences, RT-PCR was used to test if they were derived from true transcripts. Of the 48 tested 3′ ESTs of poly A+, poly A-, and bimorphic subgroups, 35 were detected in HeLa RNA (Figure S2A, Table S9), and 39 were detected in human fetal brain, kidney and liver RNA (Figure S2B). Although certain sequences could be produced by experimental artifacts, including non-specific PCR amplification, 454 sequence error and “homopolymer” sequences inherited with the pyrosequencing used by the 454 system [17], the verification results suggest that many unmapped 3′ ESTs were originated from authentic transcripts. A possible source could be related to the differences between the HeLa genome and the human genomes that contributed the human genome reference sequences. HeLa cells were derived from cervical cancer cells and have adapted to *in vitro* cultural conditions for over 50 years, resulting in a genome substantially different from the normal human genomes, as reflected by its aneuploidic 70 to 164 chromosomes (http://www.atcc.org/common/catalog/numSearch/numResults.cfmatccNumCCL-2). Indeed, our analysis of genome structure in a cancer cell line Kasumi-1 shows that cancer genome sequences are substantially different from the normal human genome sequences [23]. The transcripts expressed from the unique contents in the HeLa-specific genomic DNA would not expect to map to the human genome reference sequences.

Despite the long-term indirect evidence for the presence of poly A- transcripts in eukaryotic cells, only the histone poly A- transcripts have been systematically identified at the sequencing level. Unlike the poly A+ transcripts that can be easily isolated by binding to the 3′ poly A tail using the oligo dT, isolating poly A- transcripts with no known consensus sequences is technically difficult. The Total RNA Detection system developed in this study provides a solution to overcome this obstacle. By combining with the new next-generation sequencing platforms, this system should be useful for comprehensive poly A- transcript identification. Many fundamental questions remain to be answered, including what type(s) of RNA polymerase generates the poly A- transcripts, how the poly A- transcripts are processed, whether the poly A- transcripts code for protein or they are non-coding transcripts, and more importantly, what their functions are. Answers to these questions should have significant impacts on decoding the transcriptome, annotating the transcribed elements in the genomes, and studying the biological role of poly A- transcripts.

## Materials and Methods

### RNA preparation

HeLa cells (ATCC CCL-2) were cultured in MEM medium containing 10% fetal calf serum. Cells at exponential growth were harvested with trypsin treatment. Cytoplasmic RNA was isolated from the cells by using the RNeasy midi kit (Qiagen) following the manufacturer's protocol. To ligate the RNA adaptor (5′ P-UUAAUGGUAUCAACGCAGAGUGG (ddC) -3′) to the 3′ end of all RNA templates, RNA and adaptor were mixed at an approximate 1∶10 molar ratio (50 µg RNA and 1 µl of 1,000 µM of adaptor) in a total volume of 21 µl. The mixture was heated at 75°C for 5 minutes and cooled on ice, and the following items were added to the mixture: 2.5 µl of 10× T4 RNA ligation buffer, 1 µl of DMSO, and 1 µl (20 units) of T4 RNA ligase (New England Biolabs). The ligation mixture was incubated at 37°C for 1 hour. One µl of 0.5 M EDTA was added to the mixture to stop the reaction.

### Subtraction of ribosomal RNAs and removal of short RNAs

Subtraction was performed by using the RiboMinus Transcriptome Isolation Kit (Invitrogen) following the manufacturer's protocol. To increase subtraction efficiency, two additional probes were used, including a probe for the 18S ribosomal RNA: 5′ biotin-AGTCAAGTTCGACCGTCTTCTCAGC (location at 1884–1909, M10098), and a probe for the 28S ribosomal RNA: 5′ biotin-ACTAACCTGTCTCACGACGGTCT (location at 4493–4515, M11167). RNA and each set of probes were mixed at a 1∶10 molar ratio in a final hybridization solution (10 mM Tris-Cl pH 7.5, 1 mM EDTA, 1 M NaCl). After denaturing at 75°C for 10 minutes, the mixture was maintained at 37°C for 10 min. MagPrep® Streptavidin Beads (Novagen) were added to the mixture to remove the hybrids and the free probes. The subtracted RNA was precipitated by adding 1/10 volume of 3 M sodium acetate, and 2.5× volume of ethanol, and was maintained at −20°C for 30 minutes. RNA was collected by centrifugation and dissolved in water. To further remove short RNAs, the subtracted RNA was passed over a mini-column of the RNeasy mini kit (Qiagen). The eluted RNA was precipitated and used for cDNA synthesis.

### cDNA synthesis and 3′ ESTs collection

The enriched RNA was converted into double-strand cDNA by using a cDNA synthesis kit (Invitrogen) following the manufacturer's protocol, except for using the biotin-labeled primer based on the 3′ end adaptor sequences for the priming (5′ biotin-ATCTAGAGCGGCCGCAATGGCCACTCTGCGTTGATAC). Upon digestion of double-strand cDNA by NlaIII (New England Biolabs), the 3′ cDNA was isolated by using the MagPrep® streptavidin beads. An adaptor (sense primer: 5′-TTTGGATTTGCTGGTGCAGTACAACTAGGCTTAATAGGGACATG-3′, antisense primer: 5′-TCCCTATTAAGCCTAGTTGTACTGCACCAGCAAATCC-3′) was ligated to the 5′ end of the recovered 3′ cDNA. To integrate the 454 sequencing primer A and primer B, a 23-cycle PCR was performed by using a sense primer containing the 454 primer A and the 5′ adaptor- (5′-TTTGGATTTGCTGGTGCAGTACAACTAGGCTTAATAGGGACATG-3′, the underlined part is the 454 primer A and the rest is the 5′ adaptor), and an antisense primer containing the 454 primer B and the 3′ adaptor (5′-TCCCTATTAAGCCTAGTTGTACTGCACCAGCAAATCC-3′, underlined is the 454 primer B and the rest is the RNA adaptor ligated to the 3′ end of all RNA templates). PCR products were purified with a PCR purification kit (Qiagen), and used for 454 sequencing collection by reading from the 3′ end towards the 5′ of the 3′ cDNA templates using the 454 primer B as the sequencing primer.

### Sequence process

The following steps were used to generate non-redundant sequences: 1) only the sequences with the 3′ adaptor sequences were kept; 2) 3′ adaptor sequences were removed; 3) sequences shorter than 11 bps were eliminated; 4) the same sequences were combined; 5) sequences were separated into three groups: no A residue at the 3′ end, one A residue at the 3′ end, and more than one A residue at the 3′ end. Within each group, homologous sequences were combined at a cut-off of at least 90% identity and 90% coverage, the longest sequence of which was selected as the representative sequence; 6) the resulting sequences in each group were further manually checked if necessary to ensure the sequence quality. The sequences were deposited in the NCBI dbEST (dbEST ID 43676141-43728711).

### Comparison of 3′ ESTs with known transcript sequences

The known transcript sequences were downloaded in the following databases: RefSeq (ftp://ftp.ncbi.nlm.nih.gov/refseq/H_sapiens/mRNA_Prot/), mRNA (http://hgdownload.cse.ucsc.edu/goldenPath/hg18/bigZips/), EST (ftp://ftp.ncbi.nih.gov/repository/dbEST/). Two types of SAGE tag reference databases were used, including the SAGEmap full database that contains annotated SAGE tags based on the known human transcript sequences (http://www.ncbi.nlm.nih.gov/projects/SAGE/) and the GEO SAGE database that contains experimentally collected SAGE tags (http://www.ncbi.nlm.nih.gov/geo/). The poly A tail in the sequences was excluded for the comparison. The PatternHunter 2.0 program was used for sequence comparison with the parameter setting at e<0.1 (www.bioinformaticssolution.com/ph/, 24–26). For SAGE tag comparison, a 17-bp SAGE tag was extracted after CATG in each CATG-containing 3′ EST and matched to the reference SAGE tags.

### Mapping 3′ ESTs to genome sequences

The 3′ ESTs longer than 19 bp were used to map to the human genome reference sequences through BLAT (HG18, http://hgdownload.cse.ucsc.edu/downloads.html), with a minimal 90% coverage and 90% identity. The poly A tail in the poly A+ 3′ EST was excluded for the mapping. The intergenic region was defined as the region outside the annotated genes, and the intragenic region was defined as the region covered by the annotated genes [12]. The microRNA precursor sequences were downloaded from miRBase (http://microrna.sanger.ac.uk/sequences/, 27).

To study the evolutionary conservation of the intergenic sequences mapped by 3′ ESTs, the mapped sequences were aligned to the genomes of 17 vertebrate species (Vertebrate Multiz 17-way genome alignments, http://www.genome.ucsc.edu). For each aligned sequence, the divergence (substitution rate) between human and any of the 16 vertebrate species was calculated by the Kimura two-parameter method [28]. Although the neutral substitution rates between human and the majority of the 16 genomes are still lacking, based on the phylogenetic tree of the 17 species (http://www.genome.ucsc.edu/images/phylo/), rodents (mouse and rat) have the least divergence time with human among the 14 species, except for the chimpanzee and macaque. Since the mouse and rat genome sequence analyses show that the neutral substitution rate between rodent and human is around 0.5 substitution/site [24]–[25], a conservative 0.5 was used as the neutral substitution rate between human and any of the other 14 species except chimpanzee and macaque, as they are too similar to human. For each sequence comparison, the number of substitutions observed after correction for multiple hits (*O*) and the number of substitutions expected at the neutral evolution (λ) were calculated. The probability that a sequence is under conservation constraints is determined by 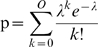.

### Experimental verification

A group of novel poly A-, poly A+, and bimorphic 3′ ESTs mapped to the human genome sequences was selected for PCR verification (Table S7). The sense primer was designed upstream of the mapped genomic location, and the antisense primer was designed based on the 3′ end sequence of each 3′ EST. HeLa RNA was used for cDNA synthesis by using MMLV reverse transcriptase (Invitrogen), and oligo dT_17_ primer or random hexamer primer. Six known poly A+ transcript sequences were used as positive control. A 30-cycle PCR was performed for each reaction by using the sense and antisense primers and either type of cDNA templates at 94°C for 30 s, 55°C for 30 s, and 72°C for 30 s. PCR products were visualized on agarose gels.

To verify the microRNA precursor-derived 3′ ESTs, a set of 3′ ESTs matching to the intronic microRNA precursor sequences was selected for the test (Table S4C). Sense and antisense primers were designed based on the 3′ ESTs using the Primer3 program, and random-priming generated cDNAs from HeLa RNA were used as the templates for PCR. PCR products were cloned into pGEMT vector (Promega) and sequenced with BigDye reagents (Applied BioSystems).

Two novel poly A- 3′ ESTs were used for northern blot confirmation. cDNA probes for each poly A- 3′ EST were generated by PCR amplification using random-priming generated HeLa cDNA templates (Table S7). Total RNAs from HepG2, kidney, HL-60, K-562 and HeLa were used for the test. The poly A+ transcript was depleted from each RNA sample by using oligo dT beads three times (Dynal). The poly A+ depleted RNA (20 ug) was fractionated through formaldehyde-denatured agarose gel, transferred to positively charges nylon membranes, and UV cross-linked. Probes were labeled with biotin using a random primer labeling method. Blots were pre-hybridized for 1 hour in hybridization buffer at 45°C and then hybridized with probes overnight at 45°C. Blots were washed twice with low stringency buffer at room temperature and twice with high stringency buffer at 45°C. The signals were then detected by chemiluminescent detection reagents.

RT/PCR was used to verify a group of the unmapped 3′ ESTs. Sense primers and antisense primers were designed based on each 3′ EST (Table S9). Random-priming generated cDNAs from human RNA of HeLa, fetal brain, kidney, and liver were used as the templates for PCR. PCR products were checked on agarose gels.

### Comparison of poly A- 3′ ESTs with poly A- “transfrag” of Affymetrix genome-tiling array

The poly A- “transfrag” data detected in HepG2 cells by Affymetrix 10 chromosome genome-tiling array were downloaded from (http://transcriptome.affymetrix.com/publication/transcriptome_10chromosomes/, 15). The poly A- 3′ ESTs mapped to the same 10 chromosomes were used to compare the genomic locations of the “transfrags”. The location shared by a 3′ EST and a “transfrag” is defined as overlapping.

## Supporting Information

Table S1 Sequences
(4.75 MB XLS)

Table S2 Novel poly A+ 3′ EST list.
(1.03 MB XLS)

Table S3 The 3′ end distribution of histone 3′ ESTs.
(0.11 MB XLS)

Table S4 Intron-originated microRNA precursors mapped by 3′ ESTs
(0.06 MB XLS)

Table S5 Overlapping genomic loci.
(1.80 MB XLS)

Table S6 Evolution conservation of 3′ EST mapped intergenic regions
(3.65 MB XLS)

Table S7 RT-PCR confirmation of novel 3′ ESTs.
(0.05 MB XLS)

Table S8 Overlapping to poly A-
(0.10 MB XLS)

Table S9 RT-PCR confirmation for the 3′ ESTs not mapped to the human genome sequences.
(0.03 MB XLS)

Figure S1 (0.25 MB TIF)

Figure S2 (0.21 MB TIF)
