# Supplementary figures and images for: Poly A- Transcripts Expressed in HeLa Cells
Source: PLoS One. 2008 Jul 30;3(7):e2803. doi: 10.1371/journal.pone.0002803 (PMC2481391; doi:10.1371/journal.pone.0002803)

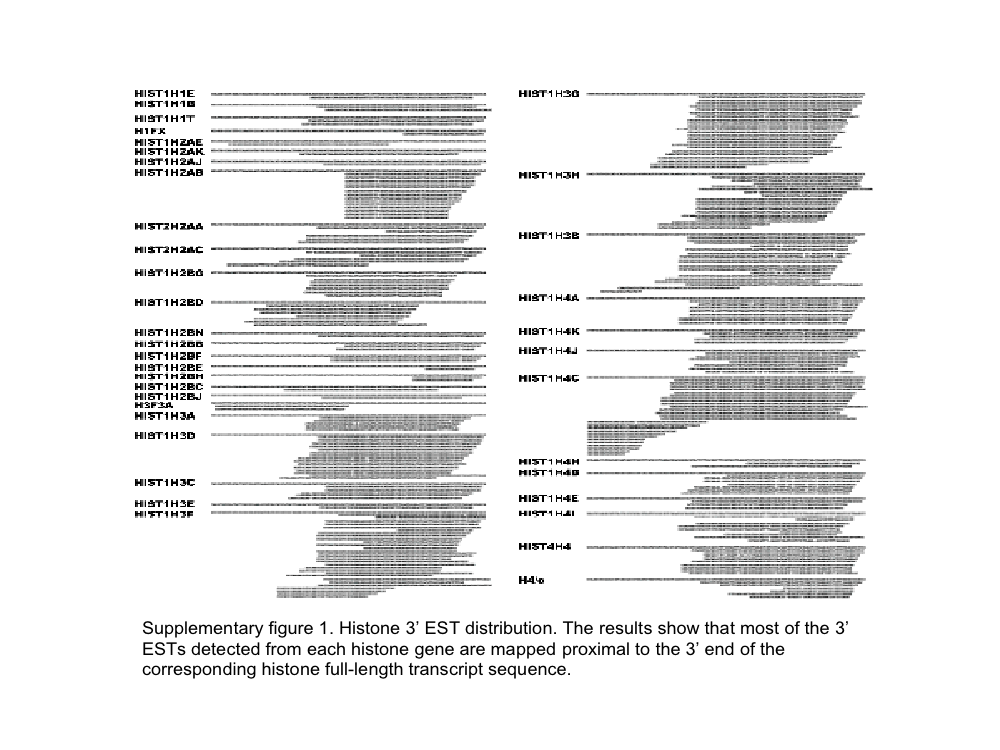

Supplement: Figure S1 — (0.25 MB TIF) [file pone.0002803.s010.tif]

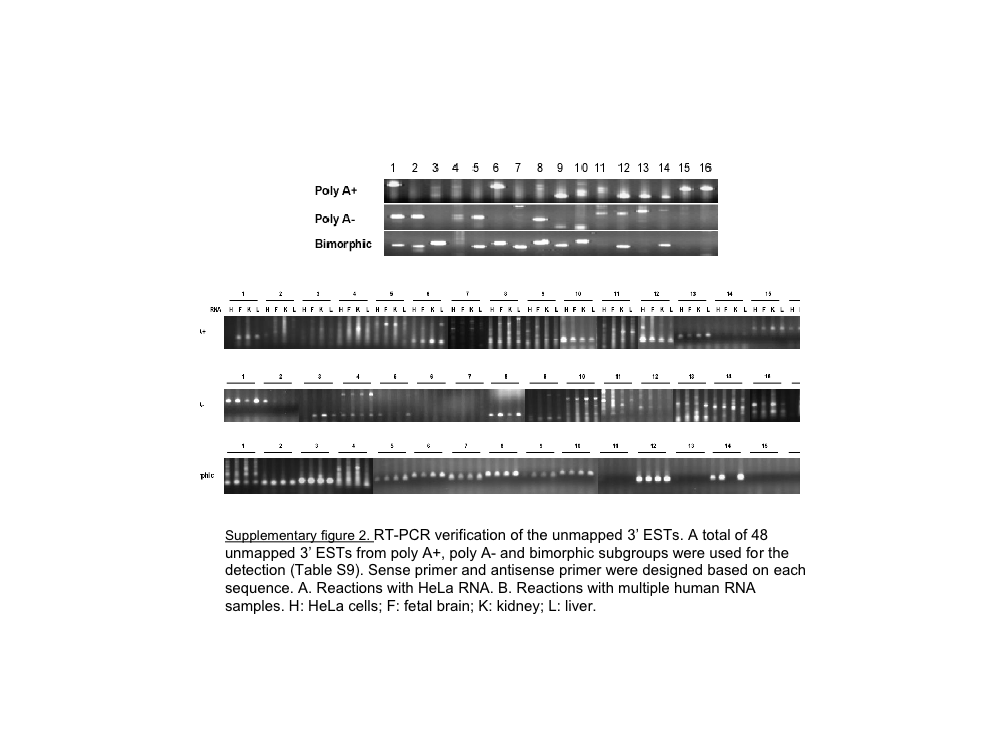

Supplement: Figure S2 — (0.21 MB TIF) [file pone.0002803.s011.tif]
